# The polymicrobial pathogenicity of *Porphyromonas gingivalis*

**DOI:** 10.3389/froh.2024.1404917

**Published:** 2024-04-26

**Authors:** Richard J. Lamont, Masae Kuboniwa

**Affiliations:** ^1^ Department of Oral Immunology and Infectious Diseases, School of Dentistry, University of Louisville, Louisville, KY, United States; ^2^ Department of Preventive Dentistry, Osaka University Graduate School of Dentistry, Suita, Osaka, Japan

**Keywords:** periodontal, community, interspecies communication, biofilm, nutritional integration

## Abstract

Accumulating microbiome data and mechanistic studies *in vitro* and *in vivo* have refined our understanding of the oral microbiota as a functionally integrated polymicrobial community. Emergent properties of these communities are driven to a large extent by interspecies communication which can be based on physical association, secreted small molecules or nutritional exchange. *Porphyromonas gingivalis* is a consensus periodontal pathogen; however, virulence is only expressed in the context of a polymicrobial community. Multivalent fimbriae mediate attachment to other oral species which can initiate a distinct transcriptional program in both constituents of the binding pair. *P. gingivalis* also responds to small molecules and nutritional cues produced by partner organisms. Physiological interdependence forms the basis of complex networks of cooperating organisms which begin to resemble an organismal entity exhibiting a spectrum of pathogenic potential.

## Introduction

Appreciation of the oral microbiome as polymicrobial harkens back to the foundation of microbiology and the meticulous observations of van Leeuwenhoek. As the new discipline of microbiology developed, however, emphasis became focused on the classical microbial diseases of the time, most of which had a single species etiology. The spectacular success of Koch's postulates which related the presence of a single exogenous organism to a single disease further entrenched the notion of individual species as etiological entities. These prevailing concepts influenced the early studies into the etiology of periodontal diseases. While it was recognized that disease was associated with an increased diversity of endogenous subgingival species, the overall goal of research was often to fulfill Koch's postulates, either in the classical form or as modified to accommodate endogenous pathogens, for periodontal microorganisms (1). This approach had some limited success with the induction of disease in non-human primates infected with *P. gingivalis* (2), albeit at a high inoculation dose unlikely to be representative of human disease. Adoption of models with extreme parameters has recently been termed, with only a hint of hyperbole, “Koch's curse” (3). Nonetheless, there was general agreement in the field that *P. gingivalis* along with a limited number of other species, functioned as periodontal pathogens (4). The introduction of the concept that complexes of organisms (color-coded depending on association with health or disease) tended to co-occur in the periodontal microbiome (5) was a significant step toward the notion of a polymicrobial etiology, particularly when integrated in a framework whereby environmental factors drive the selection and enrichment of endogenous pathogens (6). In germ-free animal models of disease, however, *P. gingivalis* remained obstinately non-pathogenic as a single species infection (7), and the idea of a polymicrobial community as the fundamental etiological unit thus took hold (8). Within this community organisms can have specialized roles, and *P. gingivalis* has emerged as a keystone pathogen which elevates overall community pathogenicity, or nososymbiocity (9), while receiving support from other community constituents which can be categorized as accessory pathogens (10). The interactions among *P. gingivalis* and other organisms that control nososymbiocity can thus define health or disease in the oral cavity to a large extent (11), and the nature of these interactions is the focus of this perspective. It is important to note, however, that periodontal tissue destruction is effectuated mainly by a dysregulated and uncontrolled inflammatory response to bacterial colonizers, a topic not addressed here but comprehensively reviewed elsewhere (12–15).

### Physical interactions

A requisite early step in the development of polymicrobial communities is inter-species adherence. Oral bacteria express a number of adhesins with specificity for genetically distinct organisms, indicating that adaptation to a polymicrobial environment has been evolutionarily selected. Study of inter-species adherence was based, for several decades, on the ability of partner organisms to clump or coaggregate in suspension, and this approach revealed complex networks of co-adherence (16, 17). In these assays, however, *P. gingivalis* displayed a very limited range of binding partners, predominantly fusobacteria. Such data contributed to the idea that *Fusobacterium nucleatum* was a bridging organism, linking so-called “late” colonizers such as *P. gingivalis* with “early” colonizers including streptococci and actinomyces (18). The development of more sensitive assays, involving adherence of *P. gingivalis* to substrates of partner species on a solid support, demonstrated that *P. gingivalis* can in fact adhere to a number of other organisms including streptococci, actinomyces, veillonellae, and treponemes (19–23). Importantly, image analyses of ex-vivo biofilm samples did not substantiate a major role for *F. nucleatum* as a physical bridging organism (24), and its role may relate more to metabolic integration (discussed below). Further, more recent evidence indicates that colonization of the tooth surface may not follow a rigid temporal progression of early, middle and late arrivals, but rather polymicrobial aggregates are preformed in saliva prior to surface attachment (25).

*P. gingivalis* deploys two, at least, types of fimbriae, comprising the structural subunit proteins FimA (major fimbriae) or Mfa1 (minor fimbriae), which account for most of the interspecies adherence activity (26). While both fimbriae also possess an accessory protein complex, the structural proteins themselves possess adhesin domains (26). FimA binds to glyceraldehyde 3-phosphate dehydrogenase (GAPDH) on streptococcal cell surfaces, as well as to the surface of *Actinomyces oris* (19, 27). In the case of GAPDH, a C-terminal domain of FimA binds to amino acid residues 166–183 of GAPDH (28, 29). Mfa1 also mediates attachment to streptococci, and a cleft spanning the central to C-terminal region of Mfa1 interacts with the SspA/B major surface proteins, with specificity being defined by the amino acid sequence of a protruding BAR domain at the C-terminus (30–32).

While close physical association between organisms facilitates small molecule or nutrition interactions, there is evidence that bacterial cells can sense and respond to the binding event itself. Outer membranes can sense stressors including mechanical changes resulting from adhesion (33). In *E. coli* for example, a surface lipoprotein can sense surface adhesion and activates two-component systems, which initiate an adhesion-dependent pattern of gene expression (34–36). Although the mechanisms have not been defined, a number of studies have shown that washed non-growing cells of *P. gingivalis* exhibit dramatic differential expression of mRNA and proteins when physically associated with other species. Contact with *T. denticola* upregulates the expression of *P. gingivalis* adhesins and proteases (37), and the organisms are synergistically pathogenic in murine models of periodontal disease (38). Time-coursed RNA-Seq showed that genes encoding a number of potential virulence determinants in *P. gingivalis* had higher expression in the presence of *S. gordonii*, including adhesins, the type IX secretion (T9SS) apparatus, and tetratricopeptide repeat (TPR) motif proteins. In contrast, genes encoding conjugation systems and many of the stress responses showed reduced expression (39). Expression of *fimA* is also increased, and stress-associated genes decreased, by *Streptococcus oralis* (40), and genes encoding T9SS components are upregulated in communities with *Candida albicans* (41). Proteomic analyses also show that partner species such as *S. gordonii* and *F. nucleatum* provide metabolic support to *P. gingivalis* in heterotypic communities (42–44), as is discussed further below. Collectively, this body of work is consistent with the notion of *P. gingivalis* virulence emerging as part of an evolutionary adaptation to a polymicrobial environment.

### Small molecule and chemical effectors

Many bacterial species utilize Autoinducer (AI)-2, an indirect product of the LuxS enzyme, to coordinate behavior, particularly in the context of homo- or hetero- typic biofilm communities (45, 46). *P. gingivalis* possesses LuxS and produces AI-2 without which heterotypic community formation with *S. gordonii* does not occur (47). Expression of *luxS* is transcriptionally upregulated in communities with *S. oralis* (40), and AI-2 may participate in a positive feedback loop controlling expression of the Mfa1 fimbrial adhesin (48). The precise role of AI-2/LuxS is difficult to discern, however, as *P. gingivalis* lacks a recognized receptor for detection of extracellular AI-2. S-adenosyl methionine, a precursor of AI-2, is a product of the one carbon metabolism (OCM) pathway, and this may provide a link with pathogenicity, as discussed further below. Additionally, growth and virulence of *P. gingivalis* require a non-AI endogenous diffusible small molecule. However, *P. gingivalis* can overcome this requirement by utilizing a soluble molecule provided by *V. parvula* (49).

Many species of oral streptococci produce the reactive oxygen species (ROS) hydrogen peroxide which can damage both DNA and proteins, and as such is toxic to other organisms (50). *P. gingivalis* is a fairly aerotolerant anaerobe and can mount a vigorous protective oxidative stress response (51, 52), which is particularly effective in the context of a community with other aerotolerant organisms such as *Filifactor alocis, F. nucleatum* and *Aggregatibacter actinomycetemcomitans* (53–55). However, as a reducing environment is required for gingipain activity, in order to maintain the cysteine in the catalytic domain, oxidation by hydrogen peroxide can impair gingipain function in the absence of an effect on bacterial viability (56). Perhaps unsurprisingly, the toxic action of hydrogen peroxide is scale dependent, with toxicity being lost at longer distances between organisms (57, 58). Indeed, at longer distances peroxide may enhance *P. gingivalis* pathogenicity through the oxidation of oxyhemoglobin (oxyHb) to methemoglobin (metHb), an early step in heme acquisition (59).

### Nutritional interactions

The importance of interspecies exchange of nutrients in the metabolism of *P. gingivalis* has been recognized for some time. For example, *P. gingivalis* produces isobutyric acid which stimulates growth of *T. denticola*, and reciprocally *T. denticola* produces succinic acid which enhances growth of *P. gingivalis* (60). More recent investigations have uncovered a web of nutritional connections which drive the interdependence of community constituents and are intimately involved in both enhancing and suppressing virulence.

#### One carbon metabolism (OCM)

**Figure 1:**
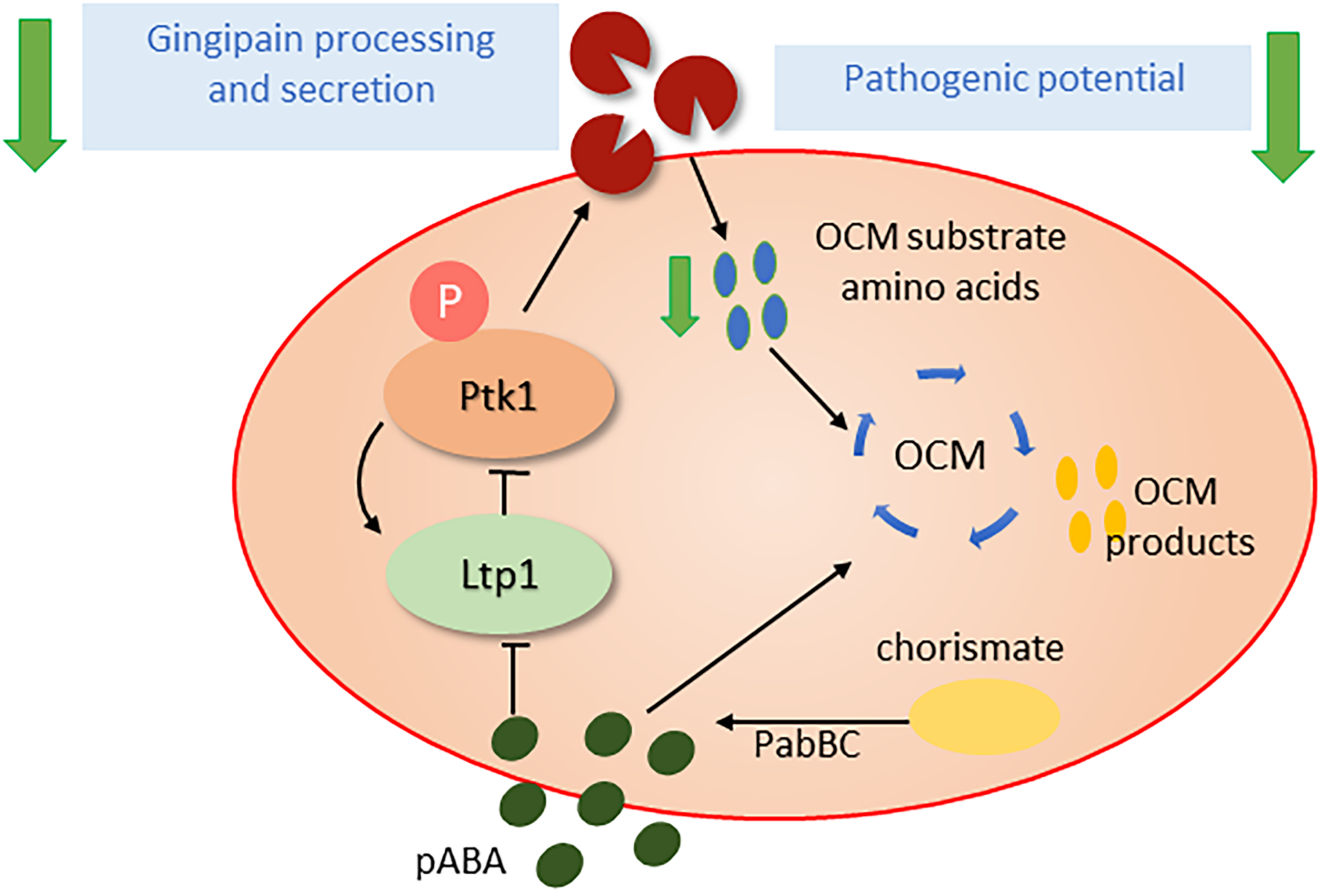
Influence of pABA (para-amino benzoic acid) on the pathogenic potential of *P. gingivalis*. pABA, an essential precursor for the one carbon metabolism (OCM) pathway, can be scavenged from partner organisms or synthesized endogenously from chorismate by the PabBC enzymes. pABA can inhibit the tyrosine phosphatase Ltp1 and thus disrupt activation state of the tyrosine kinase Ptk1 and tyrosine phosphorylation dependent signaling. Ptk1 phosphorylation of gingipains is required for optimal processing and secretion and when this is impeded the availability of amino acid substrates for OCM is diminished. Thus, the Ltp1-Ptk1 axis may function as a feedback mechanism to ensure balanced OCM flux. As gingipains are required for the provision of amino acid substrates for OCM and are major virulence factors, pABA levels regulate pathogenic potential.

OCM is an integral part of cellular intermediary metabolism, producing a number of one-carbon unit intermediates (formyl, methylene, methenyl, methyl) which are required for the synthesis of various amino acids and other biomolecules such as purines, thymidylate, folate and redox regulators (61, 62). Flux through the OCM cycle requires amino acid substrates as well as para-amino benzoic acid (pABA) which can be acquired from the extracellular milieu or produced *de novo* from chorismate by aminodeoxychorismate synthase (PabB) and 4-amino-4-deoxychorismate lyase (PabC) as part of the shikimate pathway (63). Exogenous pABA, which diffuses freely in and out of bacterial cells, can be salvaged by *P. gingivalis*, and oral streptococci such as *S. gordonii* produce exogenous pABA, which in the absence of cell-cell contact suppresses *P. gingivalis* pathogenicity. Indeed, dramatically, dual species communities of *P. gingivalis* and a *pabC* mutant of *S. gordonii* are significantly more pathogenic than a combination with the *S. gordonii* parental strain or *P. gingivalis* alone (64). The mechanisms of action of pABA remains to be fully determined; however, as an analogue of amino-benzoic acid, pABA is a competitive inhibitor of low molecular weight tyrosine phosphatases (LMW-PTPs) and consequently impacts tyrosine phosphorylation/dephosphorylation dependent signaling (65). In *P. gingivalis,* Ltp1 is a LMW-PTP and its cognate kinase, Ptk1, controls processing and secretion of gingipain proteases (66, 67), which in addition to their role as cardinal virulence factors also generate substrate amino acids for OCM flux (Figure 1). Tyrosine phosphorylation may thus serve as a metabolic couple linking OCM flux with substrate availability which in turn impacts virulence potential. Moreover, tyrosine phosphorylation based signaling in *P. gingivalis* may integrate and coordinate input from a number of organisms as Ptk1 is required for *in vivo* fitness with *F. nucleatum* (68). Further, synergistic interactions between *P. gingivalis* and other species may also involve OCM-based responses in *P. gingivalis*, as several genes in the OCM pathway are transcriptionally regulated when *P. gingivalis* is grown in *T. denticola* conditioned medium (69).

#### Regulation of biofilm life-cycle

Metabolic integration of oral organisms also impacts community lifestyle. *S. gordonii* possesses an arginine deiminase system (ADS), which catalyzes the conversion of arginine to ammonia and CO_2_, along with the production of ATP. The ADS comprises three core enzymes: ArcA, ArcB (catabolic ornithine carbamoyltransferase), and ArcC (carbamate kinase) (70). *S. gordonii* also harbors a gene encoding the arginine-ornithine antiporter (ArcD), which is commonly located in the same locus with the *arcABC* genes. ArcD, a transmembrane protein, mediates the uptake of arginine and the concomitant export of ornithine in an ATP-independent manner, thus providing a substrate for the ADS pathway. Exported ornithine induces physiological and morphological alterations of planktonic *F. nucleatum* cells and the development of heterotypic biofilms with *S. gordonii*. Thus, *S. gordonii* ArcD modulates not only alkali and energy production, but also interspecies interactions with *F. nucleatum*, initiating a middle-stage of periodontopathic biofilm formation by metabolic cross-feeding (71).

**Figure 2:**
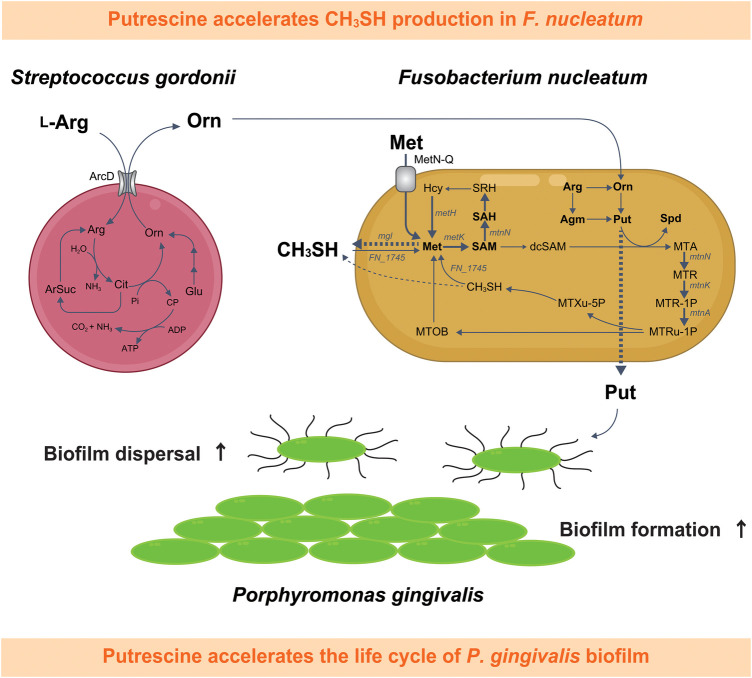
Schematic depiction of the metabolic interaction between *Fusobacterium nucleatum* and *Streptococcus gordonii,* highlighting the impact of putrescine, a metabolic byproduct synthesized during this interaction, on the life cycle of *Porphyromonas gingivalis* biofilms and CH_3_SH methyl mercaptan production in *F. nucleatum*. This diagram illustrates the pathway beginning with the uptake of L-arginine by *S. gordonii*, followed by the extracellular release of ornithine,which is subsequently metabolized *by F. nucleatum* into polyamines such as putrescine and spermidine. Putrescine released by *F. nucleatum* influences the biofilm dynamics of *P. gingivalis*. Furthermore, spermidine biosynthesis from putrescine in *F. nucleatum* is accompanied by an enhanced uptake of extracellular methionine, leading to a notable increase in CH_3_SH production. Detected metabolites are shown in bold, with dashed arrows for excretion and bold arrows for confirmed upregulation of bacterial metabolism. Met, l-methionine; SAM, S-adenosyl-l-methionine; SAH, S-adenosyl-l-homocysteine; SRH, S-ribosyl-l-homocysteine; Hcy, l-homocysteine; dcSAM, S-adenosylmethioninamine; MTA, 5'-methylthioadenosine; Arg, l-arginine; Agm, agmatine; Orn; l-ornithine; Cit, citrulline; Glu, glutamate; ArSuc, arginosuccinate; Pi, inorganic phosphate; CP, carbamoyl phosphate; Put, putrescine; Spd, spermidine; MTR, 5-methylthio-d-ribose; MTR-1P, S-methyl-5-thio-d-ribose 1-phosphate; MTRu-1P, S-methyl-5-thio-d-ribulose 1-phosphate; MTXu-5P, 1-(methylthio)xylulose 5-phosphate; CH_3_SH, methyl mercaptan; MTOB, 4-methylthio-2-oxobutanoic acid.

Conversely, when cocultured with *S. gordonii*, *F. nucleatum* increases amino acid availability to enhance the production of butyrate and putrescine, a polyamine produced by ornithine decarboxylation. Coculture with *Veillonella parvula* also increases lysine availability, promoting cadaverine production by *F. nucleatum*. Interestingly, both putrescine and cadaverine can enhance *P. gingivalis* biofilm formation, and further, putrescine also stimulates biofilm dispersal (Figure 2) (71). A corollary to this is that the species composition and ratio of early colonizers within multi-species biofilms affect the polyamine profile produced by *F. nucleatum*, and the behavior of the subsequently colonizing periodontal pathogens. Analyses of clinical specimens has confirmed not only the co-occurrence of *P. gingivalis* with genetic modules responsible for putrescine production by *S. gordonii* and *F. nucleatum* in plaque samples, but also distinct salivary metabolome profiles characterized by elevated levels of cadaverine in severe periodontitis patients and putrescine in the middle stages of periodontitis (72, 73). Furthermore, ornithine emerged as the salivary metabolite most notably associated with the periodontal inflamed surface area, as indicated by its high variable importance in projection (VIP) value, which underscores its significance in constructing predictive models for periodontal disease (74). Several other metabolomic studies employing saliva or gingival crevicular fluid as clinical specimens support these findings, with polyamines and their derivatives being identified as biomarkers for gingivitis, periodontitis or dysbiotic alteration of the periodontal microbiome (75–80).

#### Ornithine and mercaptan production

The major oral odor compound methyl mercaptan (CH_3_SH) is strongly associated with both halitosis and periodontitis. CH_3_SH production stems from metabolism of polymicrobial communities in periodontal pockets and on the tongue dorsum. Using a large-volume anaerobic non-contact coculture system, *F. nucleatum* was found to be a potent producer of CH_3_SH, with synthesis stimulated by metabolic interactions with *S. gordonii* (80). Analysis of extracellular amino acids using an *S. gordonii* ArcD mutant demonstrated that ornithine excreted from *S. gordonii* is a key contributor to increased CH_3_SH production by *F. nucleatum* (80). Furthermore, a metabolic tracing analysis with ^13^C, ^15^N-methionine, as well as gene expression analysis, revealed that ornithine secreted by *S. gordonii* increased the demand for methionine through accelerated polyamine synthesis by *F. nucleatum*, leading to elevated methionine pathway activity and CH_3_SH production. Acetylated polyamines were also detected in *F. nucleatum* cells, although their presence was dependent on the levels of putrescine and spermidine, suggesting that, in excess, putrescine and spermidine may cause polyamine acetylation which will help maintain constant intracellular levels (80). Acetylation of polyamines by diamine N-acetyltransferase in *F. nucleatum* requires acetyl-CoA. In silico analysis of the acetyl-CoA (m + 1) biosynthetic pathway from ^13^C/^15^N-labeled L-methionine (m + 6) revealed only one pathway for incorporating a ^13^C into acetyl-CoA and no pathway for a ^15^N. Notably, *F. nucleatum* cannot complete this pathway alone; it requires the enzymatic reaction of glycine hydroxymethyltransferase in the one carbon pool, which is encoded by *S. gordonii* (80). It is also likely that elevated polyamine-synthesis in the context of coexistence with *S. gordonii* increases the demand for methionine, following enhancement of methionine metabolism and CH_3_SH generation.

## Discussion

While *P. gingivalis* is commonly and correctly considered a pathogen in periodontal disease, it has become increasingly apparent that, unlike classic infections of single microbial etiology, periodontitis is not caused by a single organism but rather by polymicrobial communities of endogenous microbes acting in concert. Within these communities, organisms can have specialized roles, and although *P. gingivalis* can be seen as a driver of pathogenicity it is only in the context of a synergistic polymicrobial environment. Mapping of interspecies interactions spatially, temporally, and in molecular detail has revealed the basis of the emergent overall functions that promote or destabilize periodontal tissue homeostasis. This information provides the basis for new opportunities for intervention, not to eliminate *P. gingivalis*, which is likely a futile endeavor, but rather to restrain pathogenic potential through community engineering.

## Data Availability

The original contributions presented in the study are included in the article/Supplementary Material, further inquiries can be directed to the corresponding authors.
